# 12 tips for developing trainee-led initiatives to promote recruitment to training in shortage specialties

**DOI:** 10.15694/mep.2017.000143

**Published:** 2017-08-08

**Authors:** James Fisher, Peter Brock, Nick Saxton, Mark Garside

**Affiliations:** 1 Northumbria Healthcare NHS Foundation Trust; 2 Health Education North East

**Keywords:** Post-graduate, Careers, Recruitment, Geriatric Medicine, Trainees

## Abstract

This article was migrated. The article was marked as recommended.

The healthcare needs of the global population are changing, leaving many medical specialties facing ballooning demand in the context of under-filled specialty training programmes. In this paper we draw on a synthesis of existing literature and our experiences of developing a series of initiatives to tackle the challenge of recruitment to specialty training in geriatric medicine in the UK. We propose a set of strategies that can contribute to the development and success of such initiatives and commend these to healthcare professionals, both junior and senior, who are working in so-called ‘shortage’ specialities facing recruitment challenges. A common theme throughout the twelve tips is the need to empower trainees to deliver such initiatives. Trainees’ unique insight into training programmes, coupled with their burgeoning enthusiasm for their chosen specialty, ought to be harnessed, as it offers a ripe source of potential ideas and solutions to tackle recruitment problems.

## Introduction

Junior doctors working in 21 ^st^ century healthcare systems have a greater choice of potential career pathways than ever before, with the Association of American Medical Colleges now recognising 120 distinct specialties and sub-specialties [ 1]. As medical specialism has evolved, so too have the healthcare needs of the population. It is well recognised that worldwide, the population is ageing [ 2] - thus, there are greater numbers of older people, who are the most frequent users of healthcare services [ 3]. Ideally, the distribution of the senior clinician workforce across the array of specialisms would mirror the healthcare needs of the population - however this is not the case [ 4]. In the UK for example, emergency medicine [ 5], general practice [ 6], psychiatry [ 7] and geriatric medicine [ 8] all face ballooning demand in the context of under-filled specialty training programmes. High-level government initiatives have a key role in balancing the senior clinician workforce with the needs of the population, but these may not necessarily remedy the situation if a specialty is considered to be an unattractive career option for early-career doctors.

Against this backdrop, in 2012, a group of UK trainees in geriatric medicine began to develop a series of educational initiatives for junior doctors caring for older people. Their overarching aims were to improve standards of care for older people and, critically, to promote interest and uptake into the specialty. A non-profit organisation was founded to facilitate delivery of these goals - the Association for Elderly Medicine Education (AEME). Drawing on existing literature, and on our own experiences with AEME, we propose a set of strategies that can contribute to the development and success of such initiatives. We commend these strategies to healthcare professionals, both junior and senior, who are working in so-called ‘shortage’ specialities that face similar recruitment challenges.

**Figure 1.**
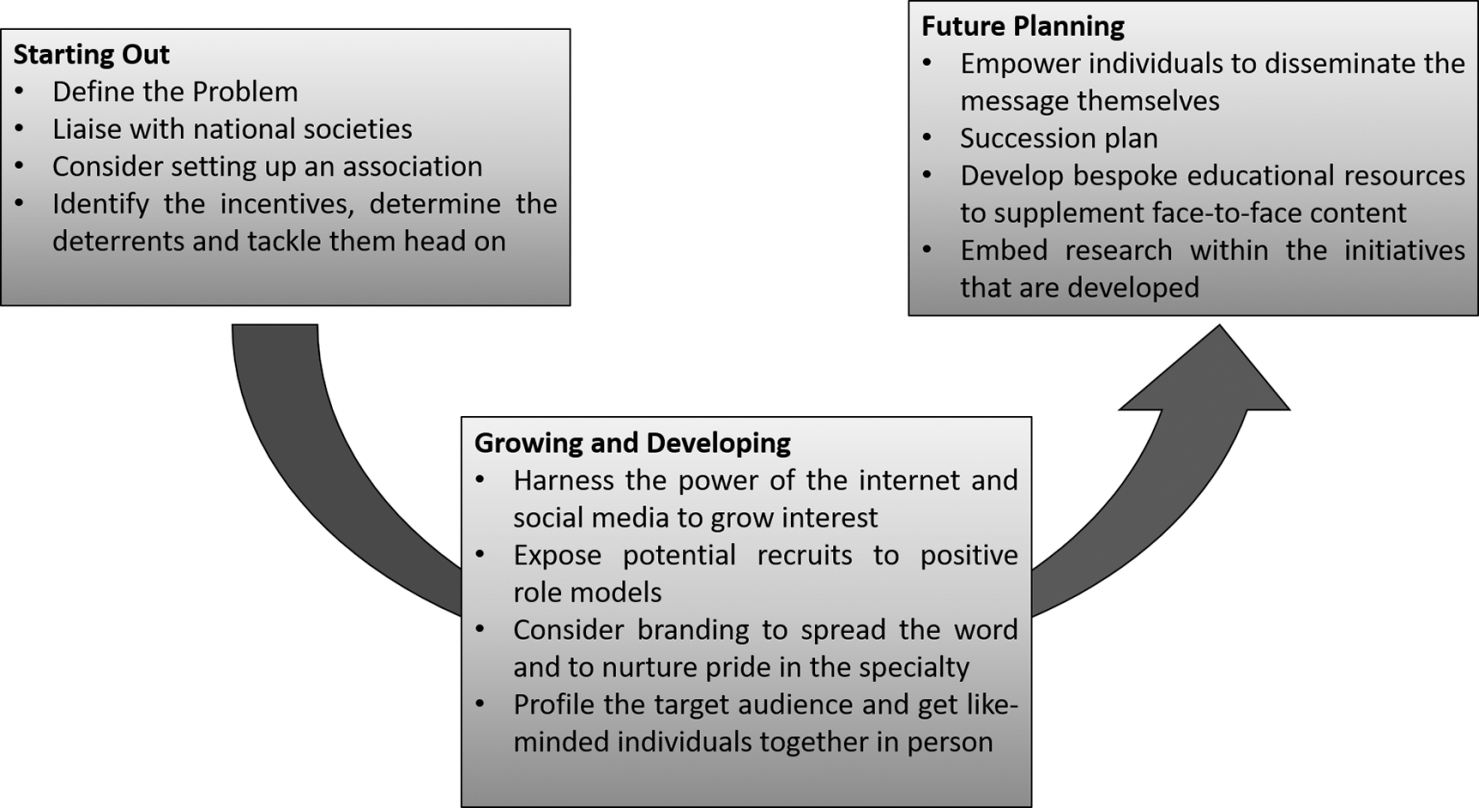
12 tips for developing trainee-led initiatives to promote recruitment to training in shortage specialties

## Starting out

### 1. Define the Problem

When starting an initiative to encourage recruitment to a shortage specialty, it is critical that the underlying issues are identified, explored and clearly articulated. It is essential that the perceived recruitment problem is confirmed as being ‘real’, and that the problem is proven to exist beyond merely your local area of work. To ensure appreciation of the ‘bigger picture’, consideration needs to be given to the needs of the population that the healthcare system serves. There should also be an appreciation of the political climate within which the healthcare system exists, as this may directly impact on the potential of recruitment initiatives. As an example of how this tip was operationalised, the first project undertaken by AEME was an exploration of the trainee workforce within the speciality of geriatric medicine.[ 8] Using year on year data obtained from national training bodies we clarified the numbers of geriatric medicine training posts available each year, applicants for each recruitment round and successful appointments. This data clearly defined the problem - the expansion in numbers of training posts in geriatric medicine, to meet the needs of the UK’s aging population, was exceeding applications, such that increasing proportions of posts were left unfilled. It was this issue that we set out to tackle.

### 2. Liaise with national societies

Early contact with national societies and training regulators is advisable. Shortage specialties may already have developed working groups to tackle the problem of under-recruitment, and early discussion may avoid unnecessary duplication of work. If such initiatives do not already exist, national bodies may be able to offer assistance and support to trainees. Such support might take the form of mentorship from a more senior colleague, or logistical support for the organisation of an educational event. National groups may also be willing to offer small-scale financial support. It is important to be mindful that involving national groups may add an additional layer of complexity to the functioning of your initiative, through the introduction of additional layers of bureaucracy. One of the advantages of working in a small-scale, trainee-led team, free from external control, is the degree of flexibility available and the speed at which decisions can be made and acted upon. This issue requires careful consideration and discussion. We contend that early, open, honest discussion with national bodies is advisable, to ensure clarity of purpose and roles. In the case of AEME, liaison with the British Geriatrics Society (the professional body of specialist doctors concerned with the health care of older people in the UK) provided invaluable logistical support, expertise in events organisation and informal mentoring from senior members of the society.

### 3. Consider setting up an Association

Depending on the scope and scale of the proposed initiative, there may be value in forming a membership organisation, set up with a particular shared purpose or aim. In the UK, this is called an unincorporated association. Formal registration or regulatory approval is not needed, and the only necessary step, by convention, is to draw up a constitution outlining how the group is to be run. This provides a collective identity to a group of individuals working together with a shared aim. Developing and formalising a constitution also commits group members to governance procedures and ensures accountability in the event of there being external critique of the initiative’s purpose or progress. The constitution informs the agenda for committee meetings, which form a key part of the association’s functioning as well as an opportunity for professional development [ 9]. Having a formal constitution provides external parties with evidence of the group’s professionalism; it also enables the group to open a bank account to deal with any necessary financial transactions. Be aware that an unincorporated association has no separate legal existence, and the members of the group are liable for any obligations or financial debts accrued.

### 4. Identify the incentives, determine the deterrents and tackle them both head on

Identifying and acknowledging the potential deterrents to recruitment to a shortage specialty is an essential step, and an early, thorough literature review is advisable. With reference to geriatric medicine, we identified some deterrents that were beyond our locus of control; for example, the limited amount of dedicated time spent at medical undergraduate level addressing geriatric medicine topics [ 10]. Other deterrents were identified that we considered amenable to direct intervention. These included negative attitudes towards older people, the perception that geriatric medicine lacks prestige [ 11] and negative perceptions about the medical registrar role (the senior training grade in UK hospital medical specialties) [ 12]. We sought to directly challenge these by organising a conference for junior doctors, which we termed ‘Geriatrics for Juniors’ (G4J). For example, to challenge the stigma of the medical registrar role we integrated a question and answer session into the conference. In this session delegates’ concerns regarding the medical registrar role could be explored and in many instances, myths about the role could be dispelled. Equally important as determining the deterrents to recruitment is identifying the incentives. At G4J we presented examples of the positive aspects of a career in the speciality, through showcasing the breadth of the sub-specialisms, highlighting the diversity of career pathways available and providing examples of the flexibility of working hours available.

## Growing and developing

### 5. Harness the power of the internet and social media to grow interest

Social media can be a powerful tool to help get your message to a wide audience at no financial cost [ 13]. The difficulty is reaching the right target audience, and getting your voice heard amongst the noise and traffic of the rest of the internet. To maximise your chance of success, we advocate creating concise and accessible content that may be more digestible amongst the morass of information online. Secondly, we would advise active engagement with like-minded individuals, since they are more likely to read and share your message, and thus increase the likelihood of it reaching the intended audience. At AEME, we used resources that were either completely free, or the free tier of a ‘freemium’ model. Twitter and Facebook were used to inform other clinicians and educators about our initiatives and we built a Wordpress website, which required minimal technical expertise, to create an online presence and to act as a hub for information about our projects. We also created short educational videos called Mini-GEMs (Geriatric E-Learning Modules), which were uploaded to YouTube - a site that is increasingly recognised as a viable platform for hosting medical education resources [ 14]. Not only have viewers found these useful for their clinical content [ 15], but they also helped to raise awareness of the organisation and its other projects.

### 6. Expose potential recruits to positive role models

Role modelling amongst medical professionals is recognised as having a powerful potential influence on the professional development of learners and also their career choices [ 16]. This influence can however be positive or negative. Positive role-modelling is recognised as an important factor underpinning why current UK geriatric medicine trainees chose a career in the specialty [ 17]. Seeking to harness the potential power of positive role-modelling, we aimed, through careful selection of the speakers at our event, to portray our specialty and the doctors working in it, as dynamic, energetic and forward-thinking. Speakers who we identified as having obvious enthusiasm and pride in the specialty, as well as their knowledge in the field were approached. However, simply placing a person who we perceived to be a positive role model behind a lectern and asking them to give a presentation, is unlikely, in isolation, to be career transforming for the listener. In an attempt to provide ongoing positive role-modelling we have latterly established an online mentoring system, OPMentor (Older Person’s Medicine Mentor), to facilitate this relationship. Speakers are invited to participate, and if agreeable, they are added to an online database of mentors. Using AEME as an intermediary, delegates can then identify and contact a given mentor for advice and support.

### 7. Consider branding to spread the word and to nurture pride in the specialty

Consideration should be given to how your specialty is being ‘sold’ to potential future recruits - does it require a ‘re-brand’? We would suggest producing a recognisable brand for your initiative, that includes a clear, eye-catching logo, as this can help to enhance both the credibility and the familiarity of your initiative amongst your target audience. Branding may also help facilitate growth of your online presence, draw people to your website and thus enable them to access your initiatives. Each of AEME’s initiatives for example, has a specific ‘brand’ that includes a unique title, logo and a clearly defined structure, such that learners know what to expect from each initiative - this reliability is recognised as a positive amongst learners [ 15]. We also provide every delegate who attends one of our events with a branded lanyard in their welcome packs. Printed on these lanyards is our website address along with text that reads “I heart geriatrics”. Delegates are encouraged to wear their lanyards in their workplace as a sign of their interest and enthusiasm for the specialty - anecdotally, these have proven to be extremely popular amongst our delegates and also amongst more senior colleagues. Such visible branding helps orientate interested people to our resources, but may also function as a ‘badge of honour’, helping nurture pride in the specialty amongst more junior doctors.

### 8. Profile the target audience and get like-minded individuals together in person

It is important to understand exactly who your intervention is targeting. In AEME’s case, our target audience were UK doctors yet to enter a specialist training post (typically doctors in the first 4-5 years post-medical school). It was evident that this group had few conferences designed specifically for their learning needs, hence the creation of G4J events. Feedback from these events highlighted the potential value that comes from bringing a group of like-minded individuals together. There is a perception amongst some junior doctors, including those who had expressed interest in geriatric medicine, that the specialty is a ‘second-class’ specialty.[ 18] Anecdotally, we found that delegates valued the opportunity to meet other doctors who shared their interest and enthusiasm for the specialty - this was, for some, empowering. Getting a large number of people with a shared interest in the same room also, for some individuals, seemed to validate their choice of geriatric medicine as a viable career option, rather than a ‘second choice’. We anticipate that similar benefits may be accessed by other shortage specialties through provision of events that bring like-minded individuals together and provide a forum for them to discuss career options.

## Future planning

### 9. Empower individuals to disseminate the message themselves

You will not be alone in your desire to attract more people to your specialty - central to producing significant change is empowering others to take up your message or intervention. It can be easy to be satisfied by improving the situation locally, but national change may be needed. There will always be limitations on your time and resources as an individual, so involving fellow trainees may enhance the potential impact of your initiative. Upscaling projects requires one to overcome natural instincts to be protective of work that has already been a success - instead of retaining control and responsibility for it, there is a need to trust others to take control and replicate it to a high standard. Secondly, empowering others requires a mechanism to be developed whereby other people who share your ideas and enthusiasm, can become involved in delivering your projects. Thirdly, easily accessible support must be available for those who take on your projects to ensure their enthusiasm does not lose momentum when challenges are encountered. An example of this tip in action is seen with AEME’s ‘G4J Connect’ events. Here, the ethos of the national G4J conference is replicated through smaller scale, local events organised by trainees outwit the AEME organisation. AEME provides logistical support, but the content, timings and location of the conference is determined by those who have approached AEME with the enthusiasm to organise an event. The result has been G4J Connect events held in 11 different cities throughout the United Kingdom, thus enabling the message to reach a much wider audience.

### 10. Succession plan

Trainees working in a shortage speciality have invaluable insight into the dilemmas of career planning, having only recently opted for a career in the specialty themselves. As such, trainees are ideally placed to craft initiatives to encourage recruitment to the specialty. We contend that such initiatives ought to be trainee-led and may lose validity in the eyes of the target audience if they are coordinated by senior clinicians with whom more junior doctors may find it harder to identify with. Similarly, senior clinicians, as time progresses, move further away from being trainees, and thus may lose a degree of insight into what it is like to be a present-day trainee. With this in mind, the founding members of AEME have, since progressing to consultancy grade, handed over the running of the association to three current trainees. It is essential for the long-term viability of such initiatives that those in charge are actively seeking talented, enthusiastic trainees who could, in time, take over the reins. Trainees, by virtue of their frequent rotations around different clinical environments, are recognised as ‘vectors’ for good practice, and as potential “agents for change” [ 19]. Whilst there is a temptation to retain a degree of control in an initiative that one has been actively involved with, it is vital that control is ceded to trainees. Failure to do so may be demotivating to incoming trainees; doing so provides trainees with freedom to innovate, but with senior supervisory support available if needed.

### 11. Develop bespoke educational resources to supplement face to face content

Whilst a one-off educational event can be a valuable learning experience, we contend that providing potential recruits with additional educational resources can help to maintain and develop burgeoning interest in the specialty. It is important to acknowledge the heterogeneity of doctors in training - what may appeal to a senior physician is unlikely to have the same impact on a newly qualified doctor. As a result, the interventions you produce cannot be ‘one size fits all’ - instead they need to be bespoke to address the learning needs of your target audience. Postgraduate, adult learning is typically self-directed, time-restricted and focussed on real-life problems [ 20]. Educational tools must therefore be time efficient, easy to access, high-quality and directly applicable to the workplace. Amongst medical educators, there is increasing support for the concept of high-quality, free, open-access ‘Meducation’ (FOAM) [ 21]. We found that developing resources that followed the principles of FOAM helped build interest and enthusiasm. For example, Mini-GEMs are short, focused, online video slideshows hosted on YouTube. Aimed at junior doctors who care for older people, they cover a variety of topics relevant to the field of geriatrics. Creating educational resources in this manner is quick and easy, and our learners valued the accessibility of the content, along with brevity and credibility of the materials presented [ 15]. Another example is CotEcast, a ‘Care of the Elderly’ podcast produced by AEME, where topics relevant to junior doctors interested in geriatric medicine are discussed and debated. The podcasts are designed to be brief, relevant to current issues and easily accessible across multiple listening platforms.

### 12. Embed research within the initiatives that are developed

We advise that trainees setting up such an initiative should aim to incorporate research into their activities. The initial literature review we undertook, to determine why trainees opted for a career in geriatric medicine, revealed limited evidence in this domain. Consequently, we aimed to integrate research alongside the education and recruitment projects we developed, and the benefits of this approach proved manifold. Firstly, we were able to answer simple questions about whether or not our interventions worked. For example, using serial surveys of delegates before and after our inaugural G4J event, we were able to demonstrate a positive impact on attitudes towards the role of the medical registrar [ 18], a key deterrent that we had hoped to tackle. Undertaking this small-scale evaluation project with senior supervision, provided the team with valuable experience. As our research experience developed, so too did our appreciation that career decisions, and the problem of under-recruitment, was a highly complex area that demanded a more nuanced research approach to better understand it. This realisation proved transformative, and led to more rigorous research, informed by an interpretative, phenomenological approach, into junior doctors’ perceptions about the role of the medical registrar [22]. Furthermore, published research on Mini-GEMs [ 15] sought to explore ‘why’ and ‘how’ learning occurred; such ‘clarification’ studies are recognised as being crucial in advancing the field of medical education research [ 23]. In sum, embedding research into AEME’s other work enabled us to better understand the problem we were seeking to address, helped inform further iterations of initiatives and also helped develop our skills and interest in the field of research.

## Conclusion

In this article we draw on our experiences of developing a series of initiatives to tackle the challenge of recruitment to UK specialty training in geriatric medicine. Given the number of specialties facing similar recruitment challenges, we commend these tips to other shortage specialties. These tips are not intended as a direct ‘recipe’ to be followed, as clearly there exists inherent differences amongst specialties, as well as variability in the training, regulatory and political climates within which they exist. Instead, we envisage educators selecting and adapting the tips that are judged to be best suited to the educational milieu in which they work. A common thread throughout the tips is the need to empower trainees to function as the agents to bring about change. Allowing trainees to have the freedom to innovate, whilst providing light-touch senior support, can act as a potent catalyst for trainees’ professional development. Trainees’ unique insight into training programmes, coupled with their burgeoning enthusiasm for their chosen specialty, ought to be harnessed, as it offers a ripe source of potential ideas and solutions to tackle recruitment problems.

## Notes On Contributors

James Fisher, MBBS MRCP MClinRes DipMedEd MD, is a consultant geriatrician and senior lecturer at Northumbria Healthcare NHS Foundation Trust. He co-founded the Association for Elderly Medicine Education (AEME).

Peter Brock, MBBS MA(Cantab) MRCP DipMedEd, is a geriatric medicine registrar at Northumbria Healthcare NHS Foundation Trust, UK. He is current chairman of AEME.

Nick Saxton, MBBS MRCP, is a geriatric medicine registrar within Health Education North East, UK. He is current treasurer of AEME.

Mark Garside, MBBS MRCP BMedSci CertMedEd MD, is a consultant stroke physician at Northumbria Healthcare NHS Foundation Trust. He co-founded AEME.
