# Supplementary material for: 12 tips for developing trainee-led initiatives to promote recruitment to training in shortage specialties
Source: MedEdPublish (2016). 2017 Aug 8;6:143. [Version 1] doi: 10.15694/mep.2017.000143 (PMC10885286; doi:10.15694/mep.2017.000143)
Supplement: Peer review history

# Reviewer report: Reviewer response for version 1

**DOI:** 10.21956/mep.19354.r28294

**Published:** 2017-10-09

**Authors:** James Read

**Affiliations:** 1 Plymouth University Peninsula Schools of Medicine and Dentistry

This review has been migrated. The reviewer awarded 5 stars out of 5 As a registrar in geriatric medicine I may of course be considered to be biased in my review of this topic! I really enjoyed reading the twelve tips and the practical approaches that can be taken to recruiting into specialties which for a long time have struggled to attract enough applicants. I'm particularly pleased to see that succession planning is included - a vital, yet often overlooked part of recruitment. Well done, a really enjoyable read.

Reviewer Expertise:

NA

No decision status is available

# Reviewer report: Reviewer response for version 1

**DOI:** 10.21956/mep.19354.r28293

**Published:** 2017-08-15

**Authors:** Trevor Gibbs

**Affiliations:** 1 AMEE

This review has been migrated. The reviewer awarded 4 stars out of 5 An interesting paper that I enjoyed reading. I specifically liked the way the authors had arranged their 12 Tips in a slightly different way to standard 12 Tips. This is an important area for development- how to recruit into areas of need. Of course Geriatrics is a necessary growth area for the future so would hope that these 12 Tips could be transferred to others specialties that are not so popular

Reviewer Expertise:

NA

No decision status is available

# Reviewer report: Reviewer response for version 1

**DOI:** 10.21956/mep.19354.r28295

**Published:** 2017-08-10

**Authors:** John Dent

**Affiliations:** 1 AMEE

This review has been migrated. The reviewer awarded 4 stars out of 5 It is always refreshing to read a new "12 Tips" paper. This approach contributes so much to encapsulating a practical approach to tackling a problem or to implementing a change.In this present paper the authors describe their experience of developing a series of initiatives to tackle the challenge of recruitment to specialty training in geriatric medicine in the UK. Their recommendations lend themselves to presentation in the "12 Tips" format and they have helpfully grouped then into three groups, Starting out; Growing and Developing, and Future Planning. A useful Figure illustrates the progress. The authors point out that these tips may be of relevance to other specialties facing similar recruitment challenges and that they are not intended as a direct ‘recipe’ to be followed.It will be interesting to see comments from reviewers in other specialties who share their concerns.

Reviewer Expertise:

NA

No decision status is available
